# On the Origin and Evolution of the Mosquito Male-determining Factor *Nix*

**DOI:** 10.1093/molbev/msad276

**Published:** 2023-12-21

**Authors:** James K Biedler, Azadeh Aryan, Yumin Qi, Aihua Wang, Ellen O Martinson, Daniel A Hartman, Fan Yang, Atashi Sharma, Katherine S Morton, Mark Potters, Chujia Chen, Stephen L Dobson, Gregory D Ebel, Rebekah C Kading, Sally Paulson, Rui-De Xue, Michael R Strand, Zhijian Tu

**Affiliations:** Department of Biochemistry, Virginia Tech, Blacksburg, VA 24061, USA; Fralin Life Sciences Institute, Virginia Tech, Blacksburg, VA 24061, USA; Department of Biochemistry, Virginia Tech, Blacksburg, VA 24061, USA; Fralin Life Sciences Institute, Virginia Tech, Blacksburg, VA 24061, USA; Department of Biochemistry, Virginia Tech, Blacksburg, VA 24061, USA; Fralin Life Sciences Institute, Virginia Tech, Blacksburg, VA 24061, USA; Department of Biochemistry, Virginia Tech, Blacksburg, VA 24061, USA; Fralin Life Sciences Institute, Virginia Tech, Blacksburg, VA 24061, USA; Department of Entomology, University of Georgia, Athens, GA 30602, USA; Center for Vector-borne Infectious Diseases, Department of Microbiology Immunology and Pathology, Colorado State University, Fort Collins, CO 80523, USA; Department of Entomology, Virginia Tech, Blacksburg, VA 24061, USA; Department of Biochemistry, Virginia Tech, Blacksburg, VA 24061, USA; Fralin Life Sciences Institute, Virginia Tech, Blacksburg, VA 24061, USA; Department of Biochemistry, Virginia Tech, Blacksburg, VA 24061, USA; Fralin Life Sciences Institute, Virginia Tech, Blacksburg, VA 24061, USA; Department of Biochemistry, Virginia Tech, Blacksburg, VA 24061, USA; Fralin Life Sciences Institute, Virginia Tech, Blacksburg, VA 24061, USA; Fralin Life Sciences Institute, Virginia Tech, Blacksburg, VA 24061, USA; Genetics Bioinformatics and Computational Biology PhD program, Virginia Tech, Blacksburg, VA 24061, USA; Department of Entomology, University of Kentucky, Lexington, KY 40503, USA; MosquitoMate, Inc., Lexington, KY 40502, USA; Center for Vector-borne Infectious Diseases, Department of Microbiology Immunology and Pathology, Colorado State University, Fort Collins, CO 80523, USA; Center for Vector-borne Infectious Diseases, Department of Microbiology Immunology and Pathology, Colorado State University, Fort Collins, CO 80523, USA; Department of Entomology, Virginia Tech, Blacksburg, VA 24061, USA; Anastasia Mosquito Control District, St. Augustine, FL 32092, USA; Department of Entomology, University of Georgia, Athens, GA 30602, USA; Department of Biochemistry, Virginia Tech, Blacksburg, VA 24061, USA; Fralin Life Sciences Institute, Virginia Tech, Blacksburg, VA 24061, USA; Genetics Bioinformatics and Computational Biology PhD program, Virginia Tech, Blacksburg, VA 24061, USA

**Keywords:** homomorphic sex chromosome, sex determination, male-determining factor, genetic engineering, mosquito control

## Abstract

The mosquito family Culicidae is divided into 2 subfamilies named the Culicinae and Anophelinae. *Nix*, the dominant male-determining factor, has only been found in the culicines *Aedes aegypti* and *Aedes albopictus*, 2 important arboviral vectors that belong to the subgenus Stegomyia. Here we performed sex-specific whole-genome sequencing and RNAseq of divergent mosquito species and explored additional male-inclusive datasets to investigate the distribution of *Nix*. Except for the Culex genus, *Nix* homologs were found in all species surveyed from the Culicinae subfamily, including 12 additional species from 3 highly divergent tribes comprising 4 genera, suggesting *Nix* originated at least 133 to 165 million years ago (MYA). Heterologous expression of 1 of 3 divergent *Nix* open reading frames (ORFs) in *Ae. aegypti* resulted in partial masculinization of genetic females as evidenced by morphology and *doublesex* splicing. Phylogenetic analysis suggests *Nix* is related to *femaleless* (*fle*), a recently described intermediate sex-determining factor found exclusively in anopheline mosquitoes. *Nix* from all species has a conserved structure, including 3 RNA-recognition motifs (RRMs), as does *fle*. However, *Nix* has evolved at a much faster rate than *fle*. The RRM3 of both *Nix* and *fle* are distantly related to the single RRM of a widely distributed and conserved splicing factor *transformer-2* (*tra2*). The RRM3-based phylogenetic analysis suggests this domain in *Nix* and *fle* may have evolved from *tra2* or a *tra2*-related gene in a common ancestor of mosquitoes. Our results provide insights into the evolution of sex determination in mosquitoes and will inform broad applications of mosquito-control strategies based on manipulating sex ratios toward nonbiting males.

## Introduction

Sex determination in insects begins with a primary signal, which is followed by intermediate signals that mediate sex-specific splicing of 2 transcription factors named *doublesex* (*dsx*) and *fruitless* (*fru*). The sex-specific isoforms of Dsx and Fru then function as the endpoint effectors that direct either male or female development (Bachtrog et al. 2014; Hopkins and Kopp 2021). The primary signals of the sex-determination cascade are highly diverse and sometimes differ between closely related species (Herpin and Schartl 2015; Saccone 2022). In the vinegar fruit fly *Drosophila melanogaster,* the double dosage of X differentiates XX from XY embryos and triggers female development (Salz and Erickson 2010). However, it is the presence or absence of a dominant male-determining factor (M factor) that distinguishes the 2 sexes in many other insects including mosquitoes. All mosquitoes belong to the monophyletic family Culicidae, which is subdivided into 2 subfamilies: the Anophelinae and Culicinae. The Anophelinae primarily consists of a single genus (*Anopheles*) with well-differentiated, heteromorphic X and Y sex chromosomes. Males are the heterogametic sex with a repeat-rich and gene-poor Y chromosome. The Culicinae consists of many, highly divergent genera that form several tribes including the: (i) Aedini which contains the genera *Aedes* and *Psorophora*; (ii) Culicini which contains the genus *Culex*; (iii) Sabethini which includes the genus *Wyeomyia*; and (iv) Toxorhynchitini which contains the genus *Toxorhynchites* (Reidenbach et al. 2009; da Silva et al. 2020; Zadra et al. 2021). In contrast to anophelines, the sex-determining chromosomes in culicines are homomorphic or autosome-like except for a pair of sex loci: the male-determining locus or the M locus, and its counterpart the m locus. Similar to *Anopheles*, the Mm male is the heterogametic sex. The M locus is approximately 1.3 Mb in length and resides on chromosome 1 (∼310 Mb) in the yellow fever mosquito *Aedes aegypti* (Matthews et al. 2018). Classic evolutionary theory suggests homomorphic sex chromosomes will eventually evolve into well-differentiated sex chromosomes through a process that involves the accumulation of sexually antagonistic genes, suppressed recombination, and consequently the decay of the heterogametic sex chromosome (Charlesworth et al. 2005; Toups and Hahn 2010; Bachtrog 2013). However, this hypothesis is the subject of recent debate (Lenormand and Roze 2022). It is not clear whether the M- and m-bearing chromosomes in the Culicinae mosquitoes represent nascent proto-Y and proto-X, respectively, or ancient sex-determining chromosomes that have maintained their homomorphic forms (Toups and Hahn 2010).

**Fig. 1.**
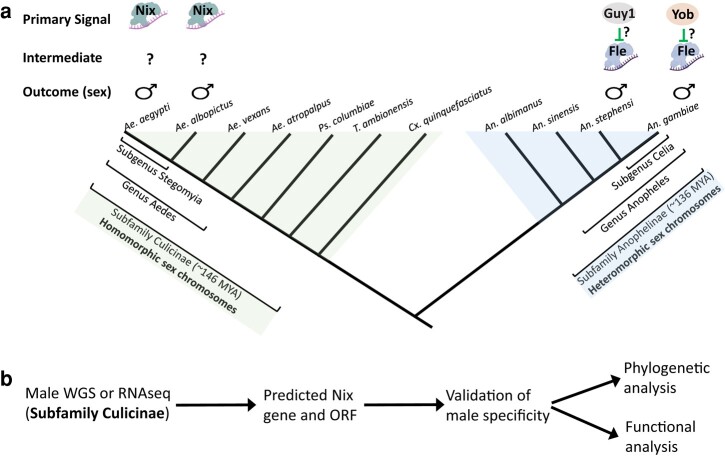
Background and workflow. a) Schematic showing known signals of the sex-determination pathway in mosquitoes. In the subfamily Culicinae, the primary signal is characterized only in 2 aedine species within the subgenus Stegomyia and it is a predicted RNA-binding protein named Nix. The presence of the primary signal Nix determines the male sex outcome, possibly through an unknown intermediate signal(s) as indicated by a question mark (Hall et al. 2015; Aryan et al. 2020). In the subfamily Anophelinae, the primary signals are characterized only in 2 anopheline species within subgenus Celia. They are Guy1 in *An. stephensi* and Yob in *An. gambiae*. Both are 56 amino acid residues in length. However, there is no significant sequence similarity between them (reviewed in Kojin, Compton et al. 2022). In *An. gambiae*, Yob somehow inactivates Fle, a presumed intermediate signal required for female development, resulting in the male sex (Krzyzinska et al. 2021). *fle* is present in all anopheline mosquitoes analyzed including *An. stephensi* (Krzyzinska et al. 2021). Nix and Fle are related and both are predicted RBPs with 3 RNA-recognition motifs (RRMs). Evolutionary context is provided based on the known phylogeny of a number of representative mosquito species. Times of origin of the Culicinae and Anophelinae are according to Zadra et al. (2021). Tree is not drawn to scale. Other time estimates exist and are mentioned in text. Not all genera within the 2 subfamilies are included. Anopheles is the major but not the only genus in subfamily Anophelinae. *Ps. columbiae*, *Psorophora columbiae; T. ambionensis, Toxorhynchites amboinensis; Cx. quinquefasciatus, Culex quinquefasciatus*. Protein and RNA illustrations are modified from Biorender Icons. b) Workflow of the study. WGS or RNAseq data from thirteen species in the subfamily Culicinae were used to identify *Nix* (Table 1). Details on -omics data acquisition, *Nix* discovery, and annotation are described in supplementary fig. S1, Supplementary Material online. ORF, open reading frame.

Early genetic evidence suggests that the M factor resides in an anopheline Y chromosome and a culicine M locus (Newton et al. 1974; Baker and Sakai 1976, 1979). *Nix*, an M-linked gene, fulfills the role of the M factor in *Ae. aegypti* as it is required for male development and sufficient to convert females into fertile males (Hall et al. 2015; Aryan et al. 2020) (See Fig. 1a). *Nix* encodes a predicted 288 amino acid (aa) RNA-binding protein distantly related to a conserved splicing factor transformer2 (*tra2*). A *Nix* homolog also functions as the M factor in the Asian tiger mosquito *Aedes albopictus* (Liu et al. 2020; Lutrat et al. 2022; Zhao et al. 2022). In the African malaria mosquito *Anopheles gambiae* and the Asian malaria mosquito *Anopheles stephensi*, Y-linked genes *gYG2/Yob*, and *Guy1*, respectively, encode M factors of 56 amino acids that are apparently not related to *Nix* (Criscione et al. 2016; Hall et al. 2016; Krzywinska et al. 2016) (Fig. 1a). As described earlier, these highly plastic primary signals eventually result in sex-specific splicing of the pre-mRNAs of 2 conserved transcription factors, *dsx* and *fru,* producing sex-specific Dsx and Fru protein isoforms that program sexual differentiation. The apparent turnover of primary signals is consistent with the hypothesis that sex-determination pathways evolve in an inverse pattern, with a tendency for frequent changes of the primary signals that control the conserved downstream factors (Bopp et al. 2014; Herpin and Schartl 2015).

Recently, another *tra2*-related gene named *femaleless (fle)* was found exclusively in *Anopheles* mosquitoes (Krzywinska et al. 2021) (Fig. 1a). Unlike *Nix*, *Yob*, and *Guy1*, which are primary sex-determining signals, *fle* is an intermediate sex-determining signal acting between *Yob* and the downstream effectors *dsx* and *fru*. In contrast to *Nix* which shifts *dsx* and *fru* splicing toward the male isoforms in *Ae. aegypti* (Hall et al. 2015; Aryan et al. 2020), *fle* is required for female-specific splicing of *dsx* and *fru* in *An. gambiae* and its action are blocked by *Yob* (Krzywinska et al. 2021) (Fig. 1a). In *D. melanogaster,* 2 splicing factors, the sex-specific transformer (tra) and nonsex-specific transformer-2 (tra2) form a complex that functions as the intermediate signal that controls the sex-specific splicing of *dsx* and *fru*. Thus, it is interesting that the aedine primary signal Nix and the anopheline intermediate signal Fle are both distantly related to tra2 (Hall et al. 2015; Krzywinska et al. 2021). Although tra has not been found in any mosquitoes (Saccone 2022), duplicate copies of the conserved tra2 are widely distributed in mosquitoes. Three tra2 genes have been reported in *Ae. albopictus* and 2 of them have been shown to affect ovarian development (Li et al. 2019).

To further advance our understanding of sex determination in mosquitoes, we conducted a broad survey of the distribution and function of *Nix* in the highly diverse subfamily Culicinae and investigated the evolutionary relationship between *Nix*, *fle*, and *tra2* (see Fig. 1b for a workflow). Although *Ae. aegypti* and *Ae. albopictus* belong to the same subgenus (Stegomyia), their Nix proteins are only 52% identical over 96% of the protein length. Low conservation and the presence of up to 100 kb-sized introns (Matthews et al. 2018; Liu et al. 2020) present significant challenges to identifying *Nix* homologs beyond closely related species. We overcame these challenges using genomics and transcriptomics analyses of male and female samples. Our results provide new insights into the evolution of sex determination and will inform broad applications of mosquito-control strategies based on manipulating sex ratios toward nonbiting males.

## Results

### Identification and Characterization of the *Nix* Gene in Divergent Mosquito Species

To characterize *Nix* in divergent mosquito species beyond *Ae. aegypti* and *Ae. albopictus*, we performed high coverage whole-genome sequencing (WGS) of 9 other species in the Culicinae (Table 1; supplementary table S1, Supplementary Material online) and developed a bioinformatic method to rapidly identify and characterize the *Nix* gene from unassembled WGS or RNAseq datasets (supplementary fig. S1, Supplementary Material online). This method was also used to identify *Nix* from 3 additional culicine species in male RNAseq databases previously deposited in the National Center for Biotechnology Information (NCBI) (Table 1). All newly characterized *Nix* sequences are provided in supplementary data S1, Supplementary Material online. Two isoforms were found in *Aedes atropalpus*, 1 of which retains a 75 nt intron. Four paralogous copies of *Nix* were identified in *Aedes triseriatus*. In total, *Nix* was identified and characterized from 12 additional species in 4 divergent genera *Aedes*, *Psorophora, Toxorhynchites*, and *Wyeomia*. We identified at least 1 *Nix* copy in all 14 species that was predicted to encode a full Nix protein. Duplicated copies showed signs of degeneration in *Ae. albopictus* and *Ae. triseriatus* (supplementary data S1, Supplementary Material online). In contrast, despite exhaustive searches in high coverage datasets including raw reads, *Nix* was not found in *Culex quinquefasciatus*, a representative of 1 of the early diverged lineages of the Culicinae.

**Table 1:** Genomic and RNAseq resources generated or used for *Nix* discovery

Species	Description	Database references
*Aedes (Stegomyia)* ^a^ *mascarensis*	WGS (male); WGS (female)	This work; PRJNA885905
*Aedes (Stegomyia) polynesiensis*	WGS (male); WGS (female)	This work; PRJNA885905
*Aedes (Stegomyia) riversi*	WGS (male); WGS (female)	This work; PRJNA885905
*Aedes (Aedimorphus) vexans*	WGS (male); WGS (female)	This work; PRJNA885905
*Aedes (Hulecoeteomyia) japonicus*	WGS (male)	This work; PRJNA885905
*Aedes (Protomacleaya) triseriatus*	WGS (male); WGS (female); RNAseq (male)	This work; PRJNA885905
*Aedes (Georgecraigius) atropalpus*	WGS (male); WGS (female); RNAseq (embryo)	This work; PRJNA885905
*Aedes (Ochlerotatus) detritus*	Published RNAseq (mixed larvae)	Edmunds et al. (2021); PRJNA431084
*Psorophora (Grabhamia) columbiae*	WGS (male); WGS (female); RNAseq (male)	This work; PRJNA885905
*Toxorhynchites sp.* ^b^	Published RNAseq: Identification to genus only	Kutty et al. (2018); SRR6155935.1
*Toxorhynchites amboinensis*	WGS (male); WGS (female); RNAseq (L3 instar)	This work; PRJNA885905
*Wyeomyia smithii*	Published RNAseq (454; male and female brain Illumina)	Tormey et al. (2015); Basrur et al. (2020) SRR11292923-5 (PRJNA612100)
*Culex (Culex) quinquefasciatus*	*Nix not found*	PRJNA661545

Notes: Detailed information on the sample source is provided in supplementary table S1, Supplementary Material online. In addition to morphological identification, species authentication has also been performed by comparing these datasets with sequences of known species origin.

^a^Subgenus names are in parentheses.

^b^Identification was determined to the genus level.

### *Nix* is Male-specific in Divergent Mosquito Species

Male-specificity of *Nix* sequences was first determined by Chromosome Quotient (CQ, Table 2) using previously published methods (Hall et al. 2015; Matthews et al. 2018). WGS data were available from both males and females in 8 of the culicine species we sequenced (Table 1). *Nix* in all these species is specific to males (CQ < 0.01) with the exception of *Ae. triseriatus* which has 3 male-specific (Ae.triNix1, 2, and 4) and 1 presumably autosomal paralog (Ae.triNix3, Table 2). No female WGS data were available for *Aedes japonicus,* and no male or female WGS data were available for *Aedes detritus*, 1 *Toxorhynchites* species, and *Wyeomyia smithii* (Table 1). Therefore, CQ analysis of *Nix* genes in these species was not possible. However, *Nix* showed no hits in female *W. smithii* RNAseq datasets, consistent with male-specificity. In addition, genomic DNA Polymerase Chain Reaction (PCR) was performed to confirm the male-specificity of the *Ae. atropalpus Nix* (supplementary fig. S2, Supplementary Material online). These results are consistent with *Nix* acting as the M factor in divergent species throughout the Culicinae.

**Table 2:** *Nix* is male-specific in divergent mosquito species

seq_name	seq_length	female_hits	male_hits	CQ
Ae.vex.NixORF	882	0	175	0
Ae.atr.Nix1ORF	816	0	121	0
Ae.pol.NixORF	849	0	270	0
Ae.tri.Nix1ORF	837	0	157	0
Ae.tri.Nix2ORF	831	0	101	0
Ae.tri.Nix3ORF^a^	618	260	276	0.94
Ae.triNix4ORF	321	0	23	0
Tox.amb.NixORF	858	0	125	0
Ae.riv.NixORF	849	0	125	0
Ae.mas.NixORF	867	1	116	<0.01
Ps.col.NixORF	831	1	139	<0.01

^a^This copy is likely autosomal.

### Evolutionary Conservation of *Nix* is Indicated by Three RNA-Recognition Motifs, a Conserved Intron Position, and Similar Three-dimensional Structures

**Fig. 2.**
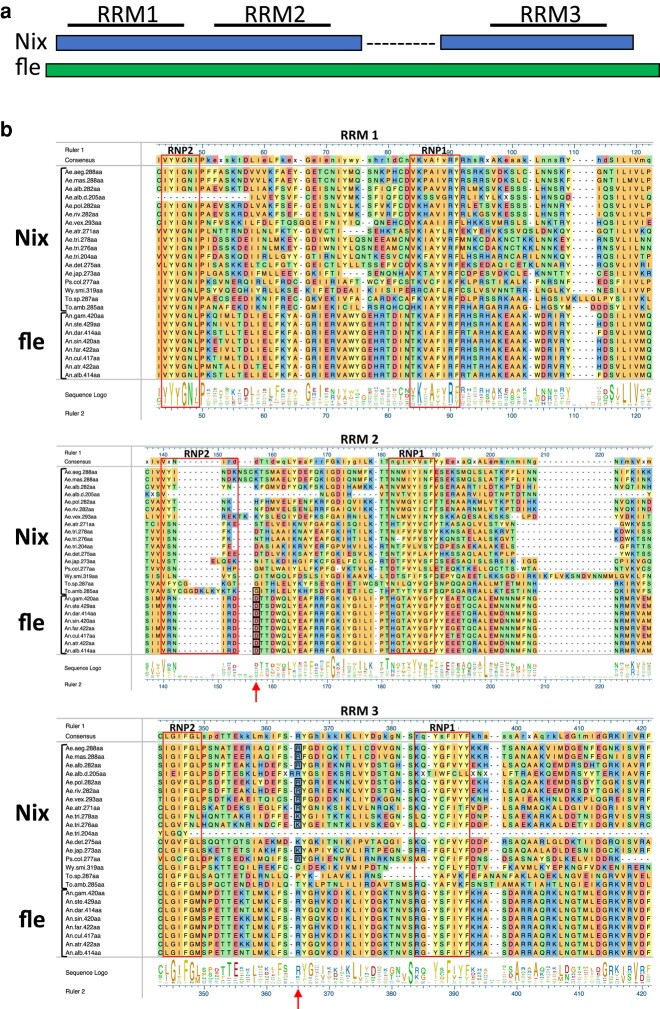
Multiple sequence alignment of *Nix* and *fle* sequences and AlphaFold structure predictions of selected *Nix* and *fle* proteins. a) Schematic shows structure of *Nix* compared to *fle*. b) Multiple sequence alignment of *Nix* and *fle*. Only RRM1, RRM2, and RRM3 are shown. Full alignment is provided as supplementary fig. S3, Supplementary Material online. Boxes surround conserved motifs RNP2 and RNP1. Arrows indicate conserved intron positions with black-boxed residues representing the codon that is split by the intron. Ae.alb.d.205aa is a degenerate copy of *Nix*, and Ae.det.275aa, Wy.smi.319aa, and To.sp.287aa are from RNAseq data only, therefore intron determination was not possible for these sequences. See Table 1 for full species’ names.

**Fig. 3.**
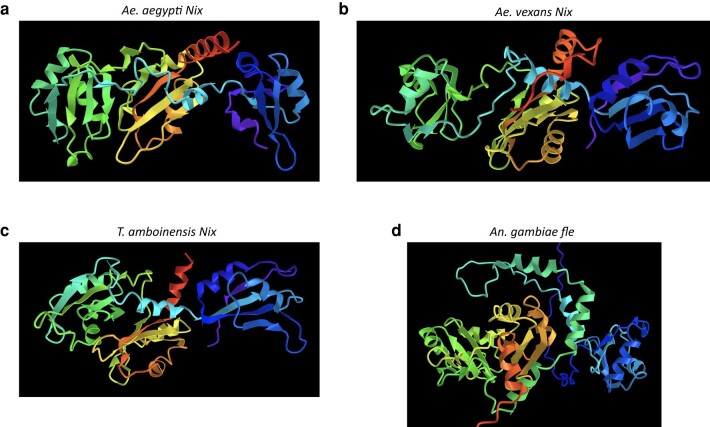
a to d) AlphaFold2 structure predictions (images from NCBI iCn3D viewer) are rainbow-colored (N-terminal red, C-terminal purple) with RRM1 (orange/yellow), RRM2 (green/turquoise) and RRM3 (blue/purple) located in center, left, and right, respectively. Some N-terminal and C-terminal disordered sequence is not shown in d.

Several characteristics of divergent Nix protein sequences indicate evolutionary conservation. All full-length Nix proteins from the 14 mosquito species we analyzed share 3 conserved RNA-binding domains (RBDs) or RNA-recognition motifs (RRMs) as previously reported for *Nix* from *Ae. aegypti* (Coronado et al. 2020) (Fig. 2). Each RRM contains the characteristic ββαββαβ secondary structure motifs and 2 short conserved ribonucleotprotein (RNP) motifs (Maris et al. 2005; Clery et al. 2008; Muto and Yokoyama 2012), RNP1 and RNP2, having 8- and 6-amino acid consensus sequences, respectively (Coronado et al. 2020). Twelve sequences from 11 species (all except *Toxorhynchites amboinensis*) had an intron in the same position in RNA recognition motif 3 (RRM3), consistent with a common origin. For 3 species only cDNA sequences are available (Table 1). Therefore, these sequences and *Nix* sequences that are from apparently degenerate paralogs were not included in the intron analysis. Finally, AlphaFold structure predictions suggested that divergent Nix proteins share similar 3-dimensional structures (Fig. 3a to d).

### *Nix* From the 14 Mosquito Species Form an Ancient Clade

**Fig. 4.**
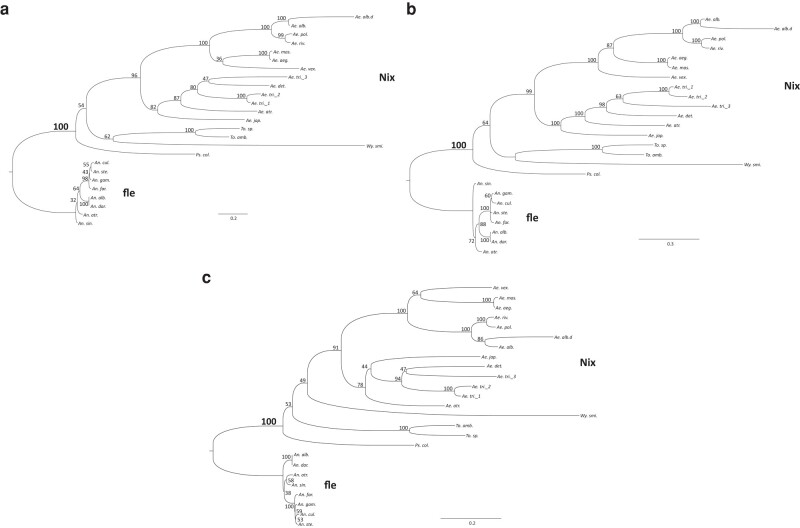
Culicinae *Nix* forms a monophyletic but highly divergent clade when rooted using *fle* as the outgroup. Phylogenetic relation of *Nix* and *femaleless* (*fle*) was inferred using a) Maximum Likelihood, b) MrBayes, and c) BOINJ. Clade credibility values are indicated. The scale bar shows substitutions per site. See supplementary fig. S3 and supplementary data S1C, Supplementary Material online for alignment used to infer phylogeny. *fle*, exclusive to Anopheline mosquitoes, was used to root the tree. *Nix* has not been found in *Culex quinquefasciatus*, a species with extensive genomic data. *Ae.alb.d* represents a degenerate copy of *Nix*. A fourth but highly truncated *Ae. triseriatus* copy (Ae.tri.4, see supplementary data S1, Supplementary Material online) was not included in this analysis. See Table 1 for full species’ names.

**Fig. 5.**
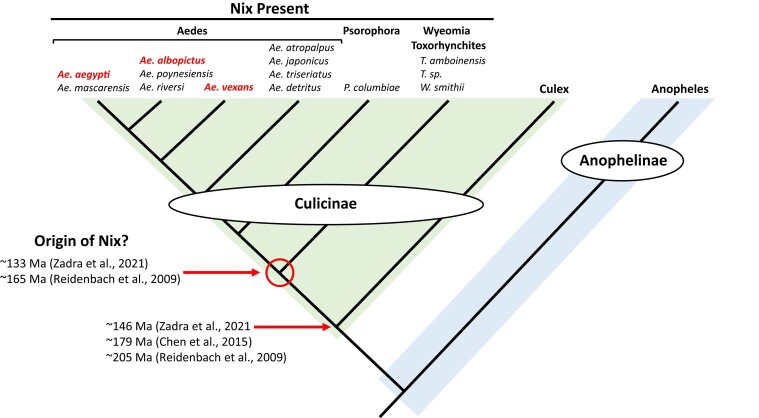
*Nix* is widely distributed and restricted to culicinae mosquitoes. Cladogram shows relationships between mosquito species based on known mosquito phylogeny (Reidenbach et al. 2009; Soghigian et al. 2017; da Silva et al. 2020; Zadra et al. 2021). Genus names are given at top and the Culicinae and Anophelinae subfamilies are shaded. Species for which *Nix* is present are indicated at the top of the figure and species for which male-determining function of *Nix* has been demonstrated are in red and bold.

As earlier noted, *Nix* is a distant homolog of the highly conserved splicing factor *tra2* (Hall et al. 2015). However, the similarity between *Nix* and *tra2* is limited to RRM3 of *Nix* and the single RRM present in *tra2* (Coronado et al. 2020; Krzywinska et al. 2021). The closest relative of *Nix*, which also has 3 RRMs (Fig. 2a and b), is a group of *Anopheles*-specific proteins named *femaleless (fle)* (Krzywinska et al. 2021). We thus aligned *fle* from divergent *Anopheles* species with the *Nix* sequences from culicine mosquitoes for comparison (Fig. 2b) and to serve as an outgroup to investigate *Nix* evolution. The entire *Nix* and *fle* alignment (Fig. 2b, supplementary fig. S3 and data S1, Supplementary Material online) was used to determine the relationship among all Nix protein sequences with *fle* serving as the outgroup for rooting the tree. We performed phylogenetic analysis using RAxML, MrBayes, and BIONJ (Fig. 4a to c) (Huelsenbeck and Ronquist 2001; Dereeper et al. 2008; Stamatakis 2014). *Nix* and *fle* formed 2 clades with high support, and sequence phylogenies were overall consistent with species phylogeny (Reidenbach et al. 2009; da Silva et al. 2020; Zadra et al. 2021). However, the precise phylogenetic groupings of *Nix* from *Psorophora, Toxorhynchites*, and *Wyeomyia* spp. were not well-supported by bootstrap or other statistical replications, perhaps due to sparse sampling and extended evolutionary divergence time among these taxa. In the Bayesian analysis (Fig. 4b), *Aedes vexans Nix* was the sister taxon of the *Nix* sequences from the subgenus Stegomyia which includes the *Ae. aegypti* and *Ae. albopictus* clades, consistent with the species phylogeny. However, maximum likelihood (Fig. 4a) and distance analysis (Fig. 4c) suggested, albeit with low statistical support, that *Ae. vexans Nix* was the sister taxon of the *Nix* sequences from the *Ae. aegypti* and *Aedes mascarenesis* clade. The *Nix* paralogs in *Ae. albopictus* and *Ae. triseriatus* are largely grouped within species, indicating duplication events occurred recently. The branch lengths (estimated amino acid substitutions) of sequences in the *Nix* clade are much longer than for sequences in the *fle* clade, indicating faster evolution of *Nix* which is remarkable given the estimated origin times of the Anopheles (94 Ma) and Aedes (105 Ma) genera (Moreno et al. 2010; Zadra et al. 2021). Furthermore, considering the estimated origin time for the *Stegomyia* subgenus of Aedes (∼73 Ma) (Zadra et al. 2021), *Nix* branch lengths in *Stegomyia* are still much longer than those of *fle*. The dn/ds ratios for *Nix* sequences from *Stegomyia* are approximately 4 times higher than those for *fle* sequences (0.26 to 0.29 vs. 0.06 to 0.07, supplementary table S2, Supplementary Material online), suggesting a lower level of purifying selection on *Nix* compared to *fle*. Other estimates of the origin of the Aedes genus and *Stegomyia* subgenus are even more recent (Soghigian et al. 2017) further emphasizing the faster evolution of *Nix* compared to *fle*. This is consistent with the emerging trend of rapid evolution of the primary signals involved in sex determination (Bopp et al. 2014; Herpin and Schartl 2015) where *Nix* is the primary sex-determining signal in culicine mosquitoes while *fle* is further downstream in the sex-determination pathway of anopheline mosquitoes. Given the distribution of *Nix* and published mosquito species divergence times (Reidenbach et al. 2009; Zadra et al. 2021), we estimate *Nix* originated ∼133 to 165 MYA (Fig. 5).

### *Ae. vexans* Nix Shows Functional Conservation in *Ae. aegypti* While *Ae. polynesiensis* and *Ae. japonicus* Nix Do Not

**Fig. 6.**
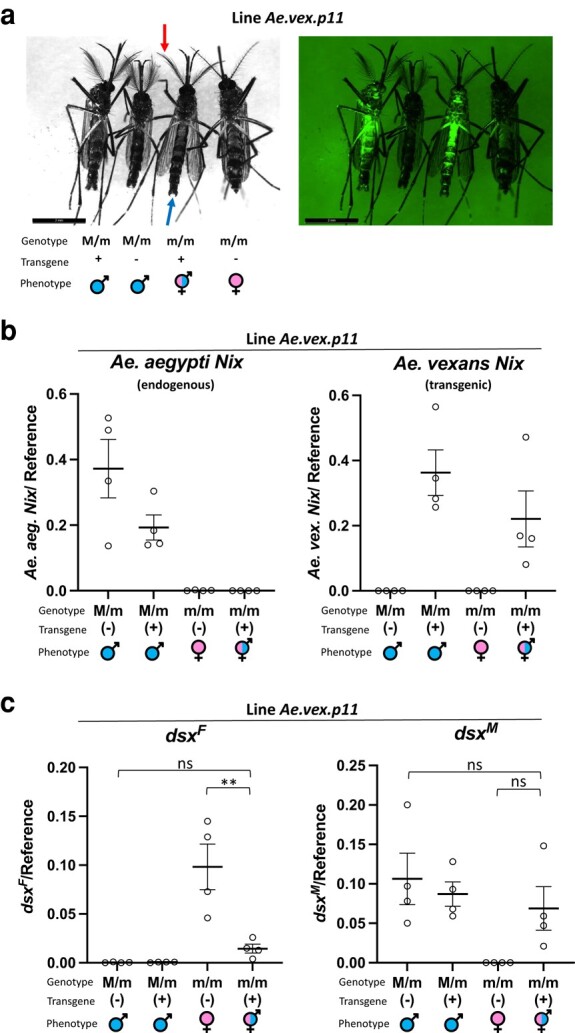
Heterologous expression of transgenic *Ae. vexans Nix* in *Ae.aegypti* masculinizes genetic females by altering the sex-determining pathway. a) Images show each of 4 genotype progeny resulting from a cross between transgenic *Ae. aegypti* males having an *Ae. vexans Nix* expression construct, and wild-type females. Image at left was captured with white light. Arrows point to typical male morphological features, plumose antennae (upper red arrow), and gonocoxites/gonostyli (lower blue arrow), that are also present in the transgenic intersex individual. Image at right was captured with fluorescent microscope using an EGFP filter, showing transgenic individuals evidenced by EGFP expression in whole body. Left panel and right panel show same individuals in same order. b) Expression of *Ae. aegypti* endogenous *Nix* (left panel) and transgenic *Ae. vexans Nix* (right panel) in *Ae. aegypti* line *Ae.vex.p11* was determined by ddPCR relative to gene AAEL002401 used as a control. Genotype, presence of transgene, and phenotype are indicated at bottom. The expression of the transgene in genetic females and males was not significantly different than the endogenous *Nix* expression in wild-type (WT) males (Tukey's multiple comparison test). c) Expression of female and male *dsx* isoforms (*dsx^F^* and *dsx^M^*) was determined by ddPCR relative to an endogenous gene AAEL002401 used as a control. Adult progeny with 4 resulting genotypes from a cross of transgenic males and wild-type females were assayed. X-axis labels: genotypic sex (male, M/m; female; m/m); ± indicates presence/absence of the *Ae. vexans Nix* transgenic cassette determined by a fluorescent marker; symbols indicate phenotypic sex (male, female, and intersex). In (b) and (c) individual values are shown with the mean and ± standard error of the mean (SEM). Statistically significant differences between transgenic genetic females and WT males/WT females are indicated from 1-way Analysis of Variance (ANOVA) followed by Tukey's multiple comparison test.

Our phylogenetic analyses consistently indicated *Nix* from *Ae. vexans* and *Ae. polynesiensis* is more closely related to *Nix* from *Ae. aegypti* than *Nix* from *Ae. japonicus* (Figs. 4 and 5). We tested the conservation of *Nix* function by introducing and expressing each in *Ae. aegypti* via *piggyBac*-mediated transformation (Handler et al. 1998). The expression cassette (supplementary fig. S4, Supplementary Material online) utilized the same *Ae. aegypti Nix* promoter/2 kb upstream sequence, 5′ and 3′ untranslated regions (UTRs) that were previously demonstrated to effectively express *Ae. aegypti Nix* and convert *Ae. aegypti* females to flightless males (Aryan et al. 2020). Transformants were selected using the pUb-enhanced green fluorescent protein (EGFP) marker in the donor cassette. In lines expressing the *Ae. vexans Nix*, various phenotypes were observed in some genetic females such as partial masculinization, flightless intersex individuals, and females that could never sustain flight (Table 3, supplementary table S3, Supplementary Material online for detailed phenotypes). We focused on line *Ae.vex.p11* which showed partial masculinization of genetic females (Fig. 6). Observed morphological features indicative of masculinization included plumose antennae and presence of gonostyli/gonocoxites, which are male-associated genitalia structures (Fig. 6a and Table 3).

**Table 3:** Transgenic lines and phenotypes

Transgenic Nix donor plasmid	#G0 families (# families with G1 positives)	Phenotype of G1 females
*187_Ae. japonicus*	13 (4)	Normal
*188_Ae. polynesiensis*	18 (7)	Normal
*189_Ae. vexans*	12 (7)	Flightless, flightless intersex, normal

Notes: All transgenic males appeared morphologically normal. The phenotypes for line 189_*Ae.vexans.Nix* was consistent for 10 generations. See supplementary table S3, Supplementary Material online for a detailed record of phenotypes.

The lack of full conversion of genetic females to males may be explained by molecular divergence of the *Ae. vexans Nix* from *Ae. aegypti Nix* and/or insufficient expression of the *Ae. vexans Nix* transgene. However, expression of *Ae. vexans Nix* in transgenic genetic females was not significantly different than *Ae. aegypti* WT *Nix* according to droplet digital PCR (ddPCR) (Fig. 6b). Reverse Transcription Polymerase Chain Reaction (RT-PCR) additionally demonstrated expression of the transgenic *Ae. vexans Nix* in only transgenic individuals (supplementary fig. S5, Supplementary Material online). *Doublesex* is a highly conserved gene at the bottom of the sex-determination pathway and is differentially spliced in males versus females (Shukla and Nagaraju 2010; Verhulst and van de Zande 2015), allowing evaluation of sex-determination signaling in individuals. We performed ddPCR to detect perturbation of sex-determination signaling in transgenic genetic females by assaying for *dsx^F^* and *dsx^M^* isoforms. As expected we detected a shift of *dsx* isforms to male-like patterns (Fig. 6c). These results show the function of *Ae. vexans Nix* has been sufficiently conserved to affect sex determination in *Ae. aegypti*.

**Fig. 7.**
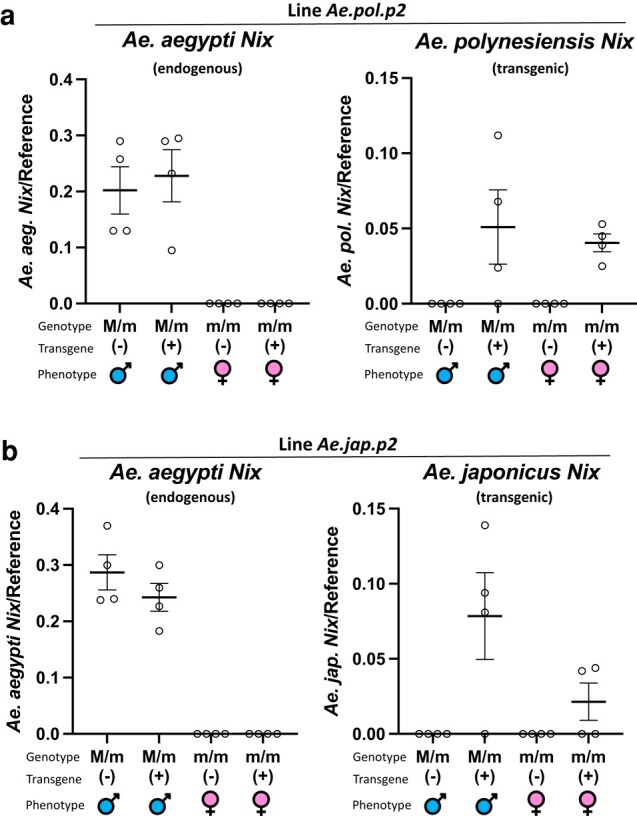
Transcription of transgenic *Ae. polynesiensis* and *Ae. japonicus Nix* compared to native *Nix*, in *Ae. aegypti* transgenic lines. a) Expression of *Ae. aegypti* endogenous *Nix* (WT) and transgenic *Ae. polynesiensis Nix* was determined by ddPCR in the *Ae. aegypti* transgenic line Ae.pol.p2, relative to gene AAEL002401 used as a control. b) Expression of *Ae. aegypti* endogenous *Nix* (WT) and transgenic *Ae. japonicus Nix* was determined by ddPCR in the *Ae. aegypti* transgenic line *Ae.jap.p2*, relative to gene AAEL002401 used as a control. In *Ae. polynesiensis* and *Ae. japonicus Nix*-expressing transgenic lines, the expression of the transgene in genetic females and males was significantly lower than the endogenous *Nix* expression in WT males according to 1-way ANOVA followed by the Tukey's multiple comparison test (*Ae. polynesiensis*, *P* = 0.0016; *Ae. japonicus*, *P* < 0.0001). Individual values are shown with the mean and ± SEM.

In contrast, multiple independent transgenic lines expressing each *Nix* open reading frame (ORF) from *Ae. polynesiensis* and *Ae. japonicus* produced no detectable phenotypes in *Ae. aegypti* (Table 3). Having multiple independent lines tends to mitigate the potential problem of position effect on transgene expression from a single insertion line. However, when we measured transcript level of the *Ae. polynesiensis* and *Ae. japonicus Nix*, transgenic *Nix* expression was significantly lower than the endogenous *Nix* as determined by ddPCR (Fig. 7). It has been suggested that an enhancer may exist in the *Nix* coding region as the *Nix* promoter, when used to drive the expression of *tetracycline transactivator* (tTA), produced significantly less tTA transcripts than the endogenous *Nix* transcripts (Kojin, Jakes et al. 2022). It is possible that the *Ae. polynesiensis* and *Ae. japonicus Nix* ORFs lack such an enhancer. Consistent with low expression of the *Ae. polynesiensis* and *Ae. japonicus Nix*, *dsx* splicing was not altered in transgenic females compared to WT (supplementary fig. S6, Supplementary Material online). However, we cannot rule out *Nix* sequence divergence being the cause of the lack of phenotype. For the *Nix* ORFs tested, their source species diverged from *Ae. aegypti* in the following order starting with the most recent (most closely related): *Ae. polynesiensis*, *Ae. vexans*, and *Ae. japonicus* (Soghigian et al. 2017) (Fig. 5). *Ae. polynesiensis* is in the subgenus *Stegomyia* with *Ae. aegypti*, whereas *Ae. vexans* is in the subgenus *Aedimorphus* (Table 1, Fig. 5). Therefore, it is surprising that a phenotype was observed for *Ae. vexans Nix* but not for *Ae. polynesiensis Nix*. Interestingly, *Ae. vexans Nix* sequence divergence does not always coincide with expectations based on source species phylogeny (Soghigian et al. 2017), as *Ae. vexans Nix* appears to be more closely related to *Ae. aegypti Nix* than *Ae. polynesiensis Nix*, in some phylogenies (Fig. 4a and c).

### *Nix* and *fle* Share a Common Origin that may Have Evolved From *tra2* or a *tra2*-related Gene in a Common Ancestor of Mosquitoes

As noted above, the closest relative of *Nix* is *fle* with both sharing 3 RRMs, while *tra2* is thought to be a distant homolog on the basis of *Nix* RRM3 sharing similarity with the single RRM present in *tra2* (Coronado et al. 2020; Krzywinska et al. 2021). *An. ga*mbiae *fle* is 420 aa while *Ae. aegypti Nix* is 288 aa but alignment showed conservation throughout the majority of each, including all 3 RRMs, which strongly suggested homology between the 2 families. However, a notable difference between them is the ∼80 aa sequence that is found in *fle* between RRM2 and RRM3, which is absent in *Nix* sequences (Fig. 2a). In search of more support for homology between *Nix* and *fle* gene families, we looked for conservation of intron position in these sequences (Fig. 2b, black-boxed residues). All analyzed *fle* genes have a conserved intron at the beginning of the coding sequence for RRM2, ranging in size from 89 to 249 nucleotides. The *Nix* sequence from *T. amboinensis* also has a predicted 152 nt intron interrupting a codon in the same position in the alignment as the intron for the *fle* sequences, supporting a common origin of *T. amboinensis Nix* and *fle* family sequences.

**Fig. 8.**
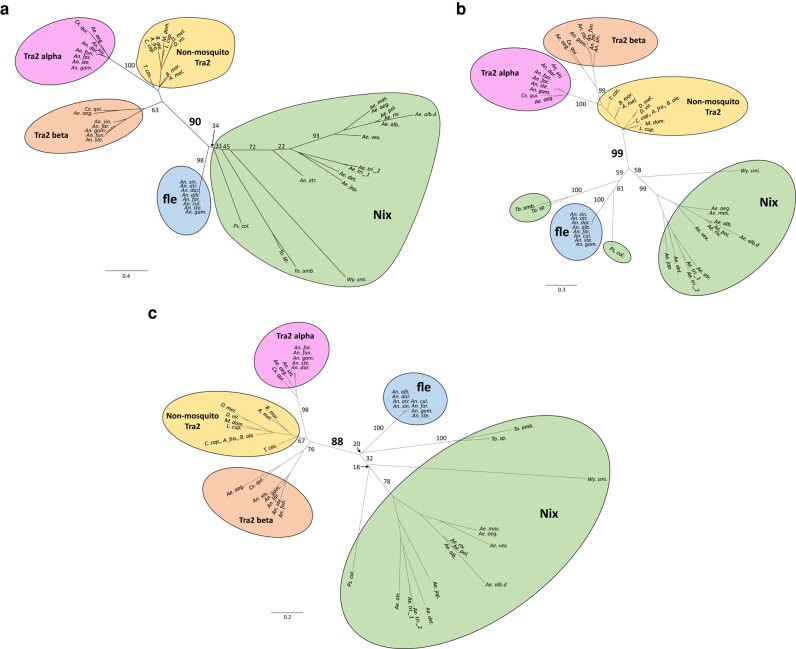
*Nix* is widespread in culicine mosquitoes and forms a sister clade to *fle* which is exclusive to anopheline mosquitoes. Shown are the unrooted phylogenies of *Nix*, femaleless (*fle*), and transformer 2 (Tra2) using amino acid sequences from RRM3 from *Nix* and *fle*, and the single RRM from Tra2. All sequences are from mosquitoes except the sequences in the “nonmosquito Tra2” clade/group. Clade credibility values are shown for major clades. The labels for clade credibility values for the branch separating Tra2 and *Nix*/*fle* clades are larger and bold for emphasis. Scale bar shows substitutions per site. See supplementary fig. S7 and data S1C, Supplementary Material online for the RRM 3 alignment used for phylogenetic inference and supplementary fig. S8, Supplementary Material online for phylogenies showing all clade credibility values. *Ae.tri._3* is not included in these trees because it does not have an RRM3. a) Maximum likelihood tree generated by RAxML using 1,000 bootstrap replicates. b) Tree generated by MrBayes with 1 million generations. *Nix* sequences from 3 species (*Ps. col, To. amb., To. sp.*) were not grouped in the *Nix* clade but are in the same bubble as all other *Nix* sequences. c) Distance tree generated by BIONJ using 1,000 bootstrap replicates. See Table 1 for full species’ names. Nonmosquito species are *Bombyx mori*, *Apis mellifera*, *Drosophila melanogaster*, *Drosophila virilis*, *Musca domestica*, *Lucilia cuprina*, *Ceratitis capitata*, *Bactrocera oleae*, *Anastrepha fraterculus*, and *Triboleum castaneum*.

To further explore potential ancestral relationships between *Nix*, *fle*, and *tra2*, we performed phylogenetic inference with RAxML, MrBayes, and BIONJ, using a multiple sequence alignment of the RRM3 domain of *Nix* (14 species) and *fle* (8 anopheline species), and the RRM domain of *tra2* sequences from mosquitoes and several other insect species (Fig. 8, alignment provided in supplementary figs. S7 and S8, Supplementary Material online). A *tra2* clade and *Nix*/*fle* clade are evident and well-supported. In contrast, our results provided only weak support for the *Nix* clade presumably due to high degree of divergence in RRM3. We also created another maximum likelihood phylogeny using only mosquito RRM3 sequences (supplementary fig. S9, Supplementary Material online) and there was still high support for *tra2* and *fle* clades, but weak support for the *Nix* clade. Yet as earlier noted, using full-length sequence with all 3 RRMs yielded very high support for *Nix* and *fle* clades (Fig. 4). Thus, our results overall support the hypothesis that *Nix* and *fle* evolved from an ancestral gene in the common ancestor of the Culicidae that subsequently diverged into their unique respective roles in sex determination in the Culicinae and Anophelinae, which have different types of sex chromosomes. The phylogeny of *tra2* homologs is also overall consistent with known insect species phylogeny, supporting multiple *tra2* duplications (*tra2-*alpha and *tra2-*beta) in the Culicidae (Li et al. 2019). Assuming *Nix*/*fle* evolved from a *tra2* or *tra2*-related ancestral sequence, these results suggest yet another duplication of *tra2* in the ancestor of mosquitoes, which subsequently evolved independently in the Culicinae and Anophelinae as *Nix* and *fle*, respectively.

## Discussion

This study identifies *Nix* family members in 14 species among 3 of 4 tribes in the subfamily Culicinae with the only exception being *C. quinquefasciatus*, which is a basal member of the subfamily. Our results suggest *Nix* originated at least ∼133 to 165 MYA on the basis of published mosquito species divergence times (Reidenbach et al. 2009; Zadra et al. 2021) (Fig. 5). Phylogenetic inference, sequence comparison, and conserved intron position all indicate that *Nix* and *fle* present in *Anopheles* spp. share a common origin (Figs. 2, 4, and 8). Neither *Nix* nor *fle* has been found in *C. quinquefasciatus*. A broader survey of other *Culex* spp. and tribes in the Culicinae will help ascertain the precise evolutionary origin of *Nix*. We hypothesize that *Culex* either lost *Nix* early in its evolution or this gene has diverged beyond recognition relative to the other culicine genera we analyzed. In this context, we note that *Nix* has evolved at a much faster rate than *fle* (Fig. 4).

Our transgenic assays indicate that *Ae. vexans Nix* can function at least partially as the M factor in *Ae. aegypti* (Fig. 6), extending functional evidence for *Nix* beyond the subgenus *Stegomyia*. However, *Nix* ORFs from *Ae. polynesiensis* and *Ae. japonicus* failed to produce detectable phenotypes in *Ae. aegypti* (Table 3, Fig. 7, and supplementary fig. S6, Supplementary Material online). As the lack of phenotype could result from insufficient transgene expression or sequence divergence in coding or other *cis*-required elements, it is important to determine the function of divergent *Nix* genes in their native species, which would likely require germline transformation (e.g. (Aryan et al. 2020)), transient embryonic injections (e.g. (Hall et al. 2015)), or manipulation of sex-specific cell lines (e.g. (Krzywinska et al. 2021)) in these species. There is at least 1 *Nix* copy in all 14 species that are predicted to encode a full Nix protein (supplementary data S1, Supplementary Material online). In addition, all *Nix* copies except a duplication in *Ae. triseriatus* have been shown to be male-specific whenever sex-specific comparisons are possible (Table 2). Thus, all current evidence is consistent with sustained *Nix* function in male mosquitoes as a male-determining factor throughout its ∼133 to 165 million years of evolution. As discussed earlier, functional verification of *Nix* sequences within their source species is necessary to determine whether or not they are indeed M factors.

We further explored the relationship between *Nix*/*fle* and their distant homolog *tra2* (Hall et al. 2015; Krzywinska et al. 2021). In divergent species from flies to humans, *tra2* is a single copy gene that produces various splice isoforms (Dauwalder et al. 1996; Beil et al. 1997; Sarno et al. 2010; Li et al. 2019). All mosquitoes have at least 2 copies of the *tra2* gene, each forming a monophyletic clade indicating that the duplication occurred in the common ancestor of the mosquito family. One of the *tra2* clades, *tra2-*beta, is evolving at a faster rate than the other *tra2-*alpha clade (Fig. 8), indicating possibly relaxed evolutionary constraints or neofunctionalization. *tra2* homology to *Nix* and *fle* is restricted to the RRM3. RRM3-based phylogenetic analysis is consistent with the hypothesis that *Nix* and *fle* evolved from another duplication of *tra2* or a *tra2*-related gene in the ancestor of mosquitoes. This hypothesis also requires the addition of 2 more RRMs in the ancestor of *Nix*/*fle*.

It is not clear how long the homomorphic sex chromosomes have been maintained in culicine mosquitoes. Although the M locus is only ∼1.3 Mb (Matthews et al. 2018) in *Ae. aegypti*, a >60 Mb M-linked region shows significant differentiation between the M- and m-bearing chromosomes (Fontaine et al. 2017; Matthews et al. 2018; Chen et al. 2022). It is currently unknown whether such a differentiation results from sex-related adaptation or simply reflects that the sex locus resides in a vast recombination desert, which is found near the centromere of all 3 chromosomes (Fontaine et al. 2017; Chen et al. 2022). Investigation into the relationship between *Nix*-linked male-determining regions across divergent species by synteny of gene blocks should provide further insights into the origin, age, and evolution of the homomorphic sex chromosomes in culicine mosquitoes. If the culicine homomorphic sex chromosomes are as old as the *Nix* gene, they will be highly attractive systems for shedding new light on how homomorphic sex chromosomes maintain homomorphy over an extended evolutionary time scale.

Genetic manipulation of the sex-determination pathway is being explored as a new way to control mosquito-borne infectious diseases as the female sex is responsible for pathogen transmission and population expansion. The identification and characterization of *Nix* in diverse mosquito species opens the door to future control applications beyond *Ae. aegypti* and *Ae. albopictus*.

## Materials and Methods

### Samples, Library Preparation, and Illumina Sequencing

Mosquito species used for high coverage WGS and RNAseq are shown in Table 1 and detailed information including sex and number of individuals are provided in supplementary table S1, Supplementary Material online. Genomic DNA was isolated using Quick-DNA Miniprep Kit (Zymo Research, Irvine, CA) or QIAamp DNA Micro Kit (Qiagen, Hilden, Germany) for pooled or single mosquito samples, respectively, according to the manufacturer's instructions. Library preparation was performed using NEBNext Ultra II FS DNA library prep kit for Illumina (New England BioLabs Inc., Ipswich, MA, USA). The quantity of the prepared DNA libraries was determined using a Qubit 2.0 Fluorometer (Thermo Fisher Scientific Inc., Waltham, MA, USA). Quality control was done by the Agilent 2100 Bioanalyzer before sequencing. RNA was isolated using either the Quick-RNA Microprep (embryonic samples) or the Quick-RNA Miniprep kit (all other samples) from Zymo Research (Irvine, CA). Libraries were prepared using the NEBNext Ultra RNA Library Prep Kit for Illumina with the Poly(A) mRNA Magnetic Isolation Module. Illumina sequencing was done either by the Virginia Tech Genomics Sequencing Center or Novogene (en.novogene.com) and the specific sequencing platform for each sample varied as indicated in submissions to the sequence read archives (SRA, PRJNA885905).

### Identifying and Assembling Nix Sequences From WGS and RNAseq Datasets

The workflow for the identification, assembly, and annotation of *Nix* in various species is summarized in supplementary fig. S1, Supplementary Material online. Further elaborating, tBLASTn (Basic Local Alignment Search Tool using protein query and translated nucleotide database) was first performed using all known Nix peptides under very low stringency (evalue 10). For example, to retrieve *Nix*-related cDNA sequences for *T. amboinensis*, larvae RNAseq data were used as the database. All *Nix*-related reads and their pairs were retrieved and assembled using Trinity 2.8.5 with default parameters (Grabherr et al. 2011). The resulting trinity assemblies were used as queries to perform a BLASTx (Basic Local Alignment Search Tool using translated nucleotide query and protein database) against a dataset of diverse RNA-binding proteins (RBPs) to remove non-*Nix* sequences that better match other related proteins. Similarly, to retrieve *Nix*-related genomic DNA sequences, male *T. amboinensis* WGS data were used as the database in a tBLASTn search with known Nix peptides as the query. All *Nix*-related gDNA reads and their pairs were retrieved and assembled using Trinity. The resulting gDNA trinity assemblies were used as queries to perform a BLASTx against diverse RBPs to remove non-*Nix* sequences. The 2 resulting *Nix* genomic DNA fragments were separated by a large intron. Therefore, a string of Ns was added between the 2 sequences to indicate the incomplete intronic sequence. Comparison between the *T. amboinensis Nix* cDNA and the gDNA sequences confirmed/defined the exon-intron boundaries. In addition to *T. amboinensis*, we generated RNAseq or cDNA data for *Ae. atropalpus, Ae. triseriatus,* and *Psorophora columbiae* (Table 1). In the case of *Ae. atropalpus,* 2 splice isoforms exist and they were subsequently confirmed by sequencing of RT-PCR products (described below). We also downloaded transcriptomic data for either male or mixed-sex samples of *Ae. detritus*, *Toxorhynchites sp*. (identification only at the genus level), and *W. smithii* for *Nix* identification (Table 1). For all remaining species, *Nix* was characterized using male WGS. Protein sequences for *Nix* genes lacking cDNA data were predicted using FGENESH+ (Solovyev 2007) using existing Nix protein sequences as the guide. All *Nix* nucleotide and protein sequences are provided in the supplementary data S1, Supplementary Material online.

### Assessing Male-Specificity of *Nix* by CQ

For the 8 species in which both male and female WGS data were available, CQ was performed using the *Nix* ORF (Table 2) as previously described (Hall et al. 2013; Matthews et al. 2018). Briefly, male and female Illumina WGS data were aligned to *Nix* ORFs using bowtie2 (https://bowtie-bio.sourceforge.net/bowtie2/index.shtml) under default conditions and CQ was calculated as the number of female hits divided by the number of male hits. In some species, only one end of the paired reads was used for each sex as the available sequences greatly exceeded the needed coverage for CQ analysis (Hall et al. 2013).

### Phylogenetic Inferences

Multiple sequence alignments (supplementary figs. S3 and S7, Supplementary Material online) using amino acid sequences (supplementary data S1, Supplementary Material online) were generated with MegAlign Pro. Version 17.2.1. DNASTAR (Madison, WI), using the Multiple Alignment using Fast Fourier Transform algorithm. Before phylogenetic inference, nonhomologous sequence in the N-terminal region of Transformer-2 sequences was removed from the alignment. Phylogenetic inference using Maximum Likelihood was performed with 1,000 bootstrap replicates using RAxML (Stamatakis 2014) as a feature of MegAlign Pro (Version 17.2.1. DNASTAR. Madison, WI). Phylogenetic inference was performed using MrBayes 3.2.7a x86_64 (Huelsenbeck and Ronquist 2001). Default parameters and the “mixed amino acid” model were implemented for 1 M generations, where the (Whelan and Goldman) amino acid substitution model (Whelan and Goldman 2001) was determined to have a posterior probability of 1.0, and minimum and maximum probabilities of 1.0 from 2 independent runs. BIONJ was performed using Phylogeny.fr (http://www.phylogeny.fr/index.cgi) with 1,000 bootstrap replicates and default parameters (Dereeper et al. 2008). See supplementary data S1, Supplementary Material online for sequences and alignments. All phylogenetic tree images were generated using FigTree v1.4.4 (http://tree.bio.ed.ac.uk/software/figtree/) except for the tree shown in supplementary fig. S9, Supplementary Material online which was generated using Iroki (https://www.iroki.net) (Moore et al. 2020).

### Plasmid Constructs

Plasmids containing the *Nix* ORF from 3 species (*Ae. polynesiensis*, *Ae. vexans*, and *Ae. japonicus*) (Table 3, see supplementary data S2, Supplementary Material online for plasmid sequences) were designed for *piggyBac*-mediated transformation and generation of mRNA for embryonic injection in *Ae. aegypti*. Each *Nix* ORF was expressed using the UTRs and promoter/upstream sequence from *Ae. aegypti* (Aryan et al. 2020). ORFs were synthesized and cloned into *piggyBac* donor backbone plasmids (Horn and Wimmer 2000) by Epoch Life Sciences (Missouri City, TX).

### Mosquito Rearing

*Ae. aegypti* (Liverpool strain) was maintained at 28 °C and 60% to 70% humidity, with a 14/10 h day/night light cycle. Adult mosquitoes were maintained on 10% sucrose and blood-fed using artificial membrane feeders and defibrinated sheep's blood (Colorado Serum Company; Denver, CO).

### *Piggy*Bac-Mediated Transformation

Donor plasmids were co-injected at 0.5 μg/μl with the *piggyBac* mRNA helper at 0.3 μg/μl into 1 h old embryos (Handler et al. 1998). Surviving G_0_ females were mated to Liverpool males in pools of 20 to 25. G_0_ males were mated individually to 5 Liverpool females and mosquitoes from 15 to 20 of these cages were merged into 1 large pool. G_1_ larvae were screened for EGFP fluorescence using a Leica M165 FC fluorescence microscope. Positive G_1_ individuals were out-crossed to Liverpool females to ensure all transgene cassettes were stably inherited to the G_2_ generation. Three *piggyBac-*based donor constructs were used: the 187_Ae. japonicus, 188_Ae. polynesiensis, and 189_Ae. vexans were injected into *Ae. aegypti* embryos (Liverpool) with *piggyBac* mRNA helper. Transgenic individuals were identified in 4 187_Ae. japonicus, 7 188_ Ae. polynesiensis, and 7 189_Ae. vexans pools. In the 189_Ae. vexans experiment, a range of phenotypes were observed including flightless intersex and flightless females.

### PCR on *Ae. atropalpus* gDNA

Genomic DNA was extracted using Zymo Quick-gDNA Miniprep Plus kit. PCR was performed with the following reaction in total volume of 20 μl: 1X Phire reaction buffer, 200 μM deoxynucleotide triphosphates (dNTPs), 0.5 μM of primers, 0.4 μl of Phire Hot Start II DNA Polymerase (Thermo Scientific), and 10 ng of gDNA. The PCR cycling condition is as following: 98 °C 30 s, followed by 30 cycles of 98 °C 5 s, 63 °C 5 s, and 72 °C 15 s, finally 72 °C 10 min.

### RT-PCR

*Ae. aegypti* adult males and females were collected and flash frozen. RNA was extracted following the total RNA extraction protocol for the Quick-RNA Miniprep kit (Zymo Research, Irvine, CA) and RNA samples were treated by DNase I. cDNA synthesis was performed using SuperScript III RT first strand cDNA synthesis kit (Invitrogen). The amount of starting RNA in each reverse transcription reaction was 2 μg. The final cDNA product was diluted 1:3 with nuclease-free water and stored at −20 °C. For RT-PCR of *Ae.vex.Nix* samples*, AevexNix* and RPS7 (reference gene) was performed using Phire II DNA polymerase (Thermo Fisher Scientific)**. The cycling conditions for vexNix and RPS7 reactions were as follows: 98 °C for 30 s; 33 cycles of 98 °C for 5 s, 55 °C for 5 s, and 72 °C for 15 s; and final extension at 72 °C for 1 min. For RT-PCR of *Ae. atropalpus Nix samples*, ∼7 male and 7 female pupae were used to isolate total RNA and cDNA was made as described above. PCR was performed with the following reaction in total volume of 20 μl: 1X Phire reaction buffer, 200 μM dNTPs, 0.5 μM of primers, 0.4 μl of Phire Hot Start II DNA polymerase (Thermo Scientific), and 10 ng of cDNA. The PCR cycling condition is as following: 98 °C 30 s, followed by 35 cycles of 98 °C 5 s, 55 °C 10 s, and 72 °C 20 s, finally 72 °C 10 min. PCR products amplified with primers Aeatro-Nix-F1/R1 were purified with GFX PCR DNA and Gel band purification kit (GE Healthcare) and cloned into pJET1.2/blunt cloning vector. One positive clone from each colony was selected and sequenced. PCR products amplified with primers Aeatro-Nix-F2/R2 were purified with GFX PCR DNA and Gel Band Purification Kit (GE Healthcare) and directly sequenced.

### RT-qPCR

Target gene and AAEL002401(reference gene) expression was quantified by RT-qPCR using Fluorescein amidite (FAM- and Hexachlorofluorescein (HEX)-labelled GoTaq Probe qPCR and RT-qPCR assay (Promega), TaqMan universal PCR master mix (Promega) and the CFX96 touch real-time PCR Detection System (BioRad Laboratories, Hercules, CA, USA). Samples were assayed using 4 biological replicates and Ct values were automatically generated by the BioRad CFX Manager 3.1 software. A Ct = 40 corresponding to the final RT-qPCR cycle was assigned to samples where no Ct value was obtained. Relative gene expression was calculated as ΔCt (*vexNix* Ct – AAEL002401 Ct). The target and cycling conditions were as follows, *Ae.pol.Nix.dsxF* and *dsxM*, *Ae.jap.Nix.dsxF* and *dsxM*: 95 °C for 3 min; 39 cycles of 95 °C for 10 s, 61 °C for 30 s.

### Droplet Digital PCR

ddPCR for target gene and AAEL002401 (reference gene) were performed with 2 Taqman probes and primer mixes, respectively. The 2 probes are labeled with different fluorescent dyes (Target-FAM, and AAEL002401-HEX) to allow detection of both in 1 reaction. ddPCR was performed on 4 biological replicates of males and females with a BioRad QX100 ddPCR machine using the recommended protocols and reagents (BioRad Labatories, Hercules, CA). All PCR reactions were initiated at 95 °C for 10 min. All PCR was performed for 40 cycles and denaturation was at 94 °C for 30 s. The target and annealing/extension conditions were as follows: *Ae.aegypti* Nix (endogenous), 56 °C for 1 min; *Ae.vex.Nix*, 58 °C for 1 min; *Ae.vex.dsxF* and *Ae. vex.dsxM*, 59 °C for 1 min; *Ae.pol.Nix*, 60 °C for 1 min; *Ae.jap.Nix*, 65 °C for 1 min. At the end of the reaction, samples were treated at 98 °C for 10 min.

### Statistics

Data were analyzed and associated graphs were generated using GraphPad Prism (v 9.3.1). Statistically significant differences between expression data were analyzed using 1-way ANOVA followed by Tukey's multiple comparison test.

### *Nix* Protein Structure Predictions

To predict *Nix* and *fle* protein structures, a modified AlphaFold2 (Jumper et al. 2021), called AlphaFold2 using MMSeqs2 (Mirdita et al. 2022), was used via a Jupyter notebook inside Google Collaboratory (https://colab.research.google.com/github/sokrypton/ColabFold/blob/main/AlphaFold2.ipynb). Structure models with the highest average predicted local distance difference test (pLDDT) scores (Model 1) were selected for structure images by exporting Protein Data Bank files. 3-D structure images were generated using iCn3D (Wang et al. 2020, 2022) (https://www.ncbi.nlm.nih.gov/Structure/icn3d/full.html).

### Selection Pressure Analysis

To compare selection pressures acting on *Nix* versus *fle*, representative *Nix* and *fle* ORF nucleotide sequences were separately codon-aligned (*Nix* vs. *Nix*, and *fle* vs. *fle*) using TranslatorX (http://translatorx.co.uk) (Abascal, et al. 2010). These codon-aligned sequences were then used in SNAP (Synonymous Nonsynonymous Analysis Program v 2.1.1) (https://www.hiv.lanl.gov/content/sequence/SNAP/SNAP.html) (Korber 2000) to calculate ds/dn, the rate of synonymous to nonsynonymous substitutions. Results are shown in supplementary table S2, Supplementary Material online where we report dn/ds, the rate of nonsynonymous to synonymous substitutions. Sequences chosen for comparison diverged at the origin of their respective clade of interest (Stegomyia subgenus for *Nix*, and Anopheles genus for *fle*).

### Primers and Probes List

Supplementary table S4, Supplementary Material online.

## Supplementary Material

msad276_Supplementary_Data

## Data Availability

Strains and plasmids are available upon request. Sequencing data are deposited and available at https://www.ncbi.nlm.nih.gov/bioproject/PRJNA885905/.

## References

[msad276-B1] Abascal F, Zardoya R, Telford MJ. Translatorx: multiple alignment of nucleotide sequences guided by amino acid translations. Nucleic Acids Res. 2010:38(suppl_2):W7–W13. 10.1093/nar/gkq291. 20435676 PMC2896173

[msad276-B2] Aryan A, Anderson MAE, Biedler JK, Qi Y, Overcash JM, Naumenko AN, Sharakhova MV, Mao C, Adelman ZN, Tu Z. Nix alone is sufficient to convert female *Aedes aegypti* into fertile males and myo-sex is needed for male flight. Proc Natl Acad Sci U S A. 2020:117(30):17702–17709. 10.1073/pnas.2001132117. 32661163 PMC7395513

[msad276-B3] Bachtrog D . Y-chromosome evolution: emerging insights into processes of Y-chromosome degeneration. Nat Rev Genet. 2013:14(2):113–124. 10.1038/nrg3366. 23329112 PMC4120474

[msad276-B4] Bachtrog D, Mank JE, Peichel CL, Kirkpatrick M, Otto SP, Ashman T-L, Hahn MW, Kitano J, Mayrose I, Ming R, et al Sex determination: why so many ways of doing it? PLoS Biol. 2014:12(7):e1001899. 10.1371/journal.pbio.1001899. 24983465 PMC4077654

[msad276-B5] Baker RH, Sakai RK. Male determining factor on chromosome 3 in the mosquito, *Culex tritaeniorhynchus*. J Hered. 1976:67(5):289–294. 10.1093/oxfordjournals.jhered.a108733. 1010930

[msad276-B6] Baker RH, Sakai RK. Triploids and male determination in the mosquito, *Anopheles culicifacies*. J Hered. 1979:70(5):345–346. 10.1093/oxfordjournals.jhered.a109271. 43343

[msad276-B7] Basrur NS, De Obaldia ME, Morita T, Herre M, von Heynitz RK, Tsitohay YN, Vosshall LB. Fruitless mutant male mosquitoes gain attraction to human odor. Elife. 2020:9:e63982. 10.7554/eLife.63982. 33284111 PMC7806257

[msad276-B8] Beil B, Screaton G, Stamm S. Molecular cloning of htra2-beta-1 and htra2-beta-2, two human homologs of tra-2 generated by alternative splicing. DNA Cell Biol. 1997:16(6):679–690. 10.1089/dna.1997.16.679. 9212162

[msad276-B9] Bopp D, Saccone G, Beye M. Sex determination in insects: variations on a common theme. Sex Dev. 2014:8(1-3):20–28. 10.1159/000356458. 24335049

[msad276-B10] Charlesworth D, Charlesworth B, Marais G. Steps in the evolution of heteromorphic sex chromosomes. Heredity (Edinb). 2005:95(2):118–128. 10.1038/sj.hdy.6800697. 15931241

[msad276-B11] Chen C, Compton A, Nikolouli K, Wang A, Aryan A, Sharma A, Qi Y, Dellinger C, Hempel M, Potters M, et al Marker-assisted mapping enables forward genetic analysis in *Aedes aegypti*, an arboviral vector with vast recombination deserts. Genetics. 2022:222(3):iyac140. 10.1093/genetics/iyac140. 36083009 PMC9630976

[msad276-B12] Clery A, Blatter M, Allain FH. RNA recognition motifs: boring? Not quite. Curr Opin Struct Biol. 2008:18(3):290–298. 10.1016/j.sbi.2008.04.002. 18515081

[msad276-B13] Coronado MA, Olivier DS, Borsatto KC, Amaral MS, Arni RK, Eberle RJ. In silico investigation of *Aedes aegypti* male-determining factor (NIX): RNA recognition motif-3, structural model and selective nucleic acid binding mode. bioRxiV 381210. 10.1101/2020.11.13.381210, 15 November 2020, preprint: not peer reviewed.

[msad276-B14] Criscione F, Qi Y, Tu Z. GUY1 confers complete female lethality and is a strong candidate for a male-determining factor in *Anopheles stephensi*. Elife. 2016:5:e19281. 10.7554/eLife.19281. 27644420 PMC5061544

[msad276-B15] da Silva AF, Machado LC, de Paula MB, da Silva Pessoa Vieira CJ, de Morais Bronzoni RV, de Melo Santos MAV, Wallau GL. Culicidae evolutionary history focusing on the Culicinae subfamily based on mitochondrial phylogenomics. Sci Rep. 2020:10(1):18823. 10.1038/s41598-020-74883-3. 33139764 PMC7606482

[msad276-B16] Dauwalder B, Amaya-Manzanares F, Mattox W. A human homologue of the Drosophila sex determination factor transformer-2 has conserved splicing regulatory functions. Proc Natl Acad Sci U S A. 1996:93(17):9004–9009. 10.1073/pnas.93.17.9004. 8799144 PMC38585

[msad276-B17] Dereeper A, Guignon V, Blanc G, Audic S, Buffet S, Chevenet F, Dufayard JF, Guindon S, Lefort V, Lescot M, et al Phylogeny.fr: robust phylogenetic analysis for the non-specialist. Nucleic Acids Res. 2008:36(Web Server):W465–W469. 10.1093/nar/gkn180. 18424797 PMC2447785

[msad276-B18] Edmunds C, Wilding CS, Robbie R. Pathogenicity and environmental tolerance of commercial and UK native entomopathogenic nematodes (*Steinernema* and *Heterorhabditis* spp.) to the larvae of mosquitoes (*Aedes aegypti* and *Ochlerotatus detritus*). Int J Pest Manage. 2021:67(3):232–240. 10.1080/09670874.2020.1731624.

[msad276-B19] Fontaine A, Filipovic I, Fansiri T, Hoffmann AA, Cheng C, Kirkpatrick M, Rasic G, Lambrechts L. Extensive genetic differentiation between homomorphic sex chromosomes in the mosquito vector, *Aedes aegypti*. Genome Biol Evol. 2017:9(9):2322–2335. 10.1093/gbe/evx171. 28945882 PMC5737474

[msad276-B20] Grabherr MG, Haas BJ, Yassour M, Levin JZ, Thompson DA, Amit I, Adiconis X, Fan L, Raychowdhury R, Zeng Q, et al Full-length transcriptome assembly from RNA-Seq data without a reference genome. Nat Biotechnol. 2011:29(7):644–652. 10.1038/nbt.1883. 21572440 PMC3571712

[msad276-B21] Hall AB, Basu S, Jiang X, Qi Y, Timoshevskiy VA, Biedler JK, Sharakhova MV, Elahi R, Anderson MAE, Chen X-G, et al A male-determining factor in the mosquito *Aedes aegypti*. Science. 2015:348(6240):1268–1270. 10.1126/science.aaa2850. 25999371 PMC5026532

[msad276-B22] Hall AB, Papathanos PA, Sharma A, Cheng C, Akbari OS, Assour L, Bergman NH, Cagnetti A, Crisanti A, Dottorini T, et al Radical remodeling of the Y chromosome in a recent radiation of malaria mosquitoes. Proc Natl Acad Sci U S A. 2016:113(15):E2114–E2123. 10.1073/pnas.1525164113. 27035980 PMC4839409

[msad276-B23] Hall AB, Qi Y, Timoshevskiy V, Sharakhova MV, Sharakhov IV, Tu Z. Six novel Y chromosome genes in Anopheles mosquitoes discovered by independently sequencing males and females. BMC Genomics. 2013:14(1):273. 10.1186/1471-2164-14-273. 23617698 PMC3660176

[msad276-B24] Handler AM, McCombs SD, Fraser MJ, Saul SH. The lepidopteran transposon vector, piggyBac, mediates germ-line transformation in the Mediterranean fruit fly. Proc Natl Acad Sci U S A. 1998:95(13):7520–7525. 10.1073/pnas.95.13.7520. 9636182 PMC22671

[msad276-B25] Herpin A, Schartl M. Plasticity of gene-regulatory networks controlling sex determination: of masters, slaves, usual suspects, newcomers, and usurpators. EMBO Rep. 2015:16(10):1260–1274. 10.15252/embr.201540667. 26358957 PMC4766460

[msad276-B26] Hopkins BR, Kopp A. Evolution of sexual development and sexual dimorphism in insects. Curr Opin Genet Dev. 2021:69:129–139. 10.1016/j.gde.2021.02.011. 33848958 PMC8364864

[msad276-B27] Horn C, Wimmer EA. A versatile vector set for animal transgenesis. Dev Genes Evol. 2000:210(12):630–637. 10.1007/s004270000110. 11151300

[msad276-B28] Huelsenbeck JP, Ronquist F. MRBAYES: Bayesian inference of phylogenetic trees. Bioinformatics. 2001:17(8):754–755. 10.1093/bioinformatics/17.8.754. 11524383

[msad276-B29] Jumper J, Evans R, Pritzel A, Green T, Figurnov M, Ronneberger O, Tunyasuvunakool K, Bates R, Zidek A, Potapenko A, et al Highly accurate protein structure prediction with AlphaFold. Nature. 2021:596(7873):583–589. 10.1038/s41586-021-03819-2. 34265844 PMC8371605

[msad276-B30] Kojin BB, Compton A, Adelman ZN, Tu Z. Selective targeting of biting females to control mosquito-borne infectious diseases. Trends Parasitol. 2022:38(9):791–804. 10.1016/j.pt.2022.05.012. 35952630 PMC9372635

[msad276-B31] Kojin BB, Jakes E, Biedler JK, Tu Z, Adelman ZN. Partial masculinization of *Aedes aegypti* females by conditional expression of Nix. PLoS Negl Trop Dis. 2022:16(7):e0010598. 10.1371/journal.pntd.0010598. 35776760 PMC9307153

[msad276-B32] Korber B . Computational analysis of HIV molecular sequences. In: Rodrigo AG, Learn GH, editors. HIV Signature and sequence variation analysis. Netherlands: Kluwer Academic Publishers; 2000. p. 55–72.

[msad276-B33] Krzywinska E, Dennison NJ, Lycett GJ, Krzywinski J. A maleness gene in the malaria mosquito *Anopheles gambiae*. Science. 2016:353(6294):67–69. 10.1126/science.aaf5605. 27365445

[msad276-B34] Krzywinska E, Ferretti L, Li J, Li J-C, Chen C-H, Krzywinski J. *Femaleless* controls sex determination and dosage compensation pathways in females of anopheles mosquitoes. Curr Biol. 2021:31(5):1084–1091.e4. 10.1016/j.cub.2020.12.014. 33417880 PMC7955153

[msad276-B35] Kutty SN, Wong WH, Meusemann K, Meier R. A phylogenomic analysis of Culicomorpha (Diptera) resolves the relationships among the eight constituent families. Syst Entomol. 2018:43(3):434–436. 10.1111/syen.12285.

[msad276-B36] Lenormand T, Roze D. Y recombination arrest and degeneration in the absence of sexual dimorphism. Science. 2022:375(6581):663–666. 10.1126/science.abj1813. 35143289

[msad276-B37] Li X, Jin B, Dong Y, Chen X, Tu Z, Gu J. Two of the three transformer-2 genes are required for ovarian development in *Aedes albopictus*. Insect Biochem Mol Biol. 2019:109:92–105. 10.1016/j.ibmb.2019.03.008. 30914323 PMC6636634

[msad276-B38] Liu P, Jin B, Li X, Zhao Y, Gu J, Biedler JK, Tu ZJ, Chen X-G. Nix is a male-determining factor in the Asian tiger mosquito *Aedes albopictus*. Insect Biochem Mol Biol. 2020:118:103311. 10.1016/j.ibmb.2019.103311. 31901476 PMC10211468

[msad276-B39] Lutrat C, Olmo RP, Baldet T, Bouyer J, Marois E. Transgenic expression of Nix converts genetic females into males and allows automated sex sorting in *Aedes albopictus*. Commun Biol. 2022:5(1):210. 10.1038/s42003-022-03165-7. 35256751 PMC8901906

[msad276-B40] Maris C, Dominguez C, Allain FH. The RNA recognition motif, a plastic RNA-binding platform to regulate post-transcriptional gene expression. FEBS J. 2005:272(9):2118–2131. 10.1111/j.1742-4658.2005.04653.x. 15853797

[msad276-B41] Matthews BJ, Dudchenko O, Kingan SB, Koren S, Antoshechkin I, Crawford JE, Glassford WJ, Herre M, Redmond SN, Rose NH, et al Improved reference genome of *Aedes aegypti* informs arbovirus vector control. Nature. 2018:563(7732):501–507. 10.1038/s41586-018-0692-z. 30429615 PMC6421076

[msad276-B42] Mirdita M, Schutze K, Moriwaki Y, Heo L, Ovchinnikov S, Steinegger M. ColabFold: making protein folding accessible to all. Nat Methods. 2022:19(6):679–682. 10.1038/s41592-022-01488-1. 35637307 PMC9184281

[msad276-B43] Moore RM, Harrison AO, McAllister SM, Polson SW, Wommack KE. Iroki: automatic customization and visualization of phylogenetic trees. PeerJ. 2020:8:e8584. 10.7717/peerj.8584. 32149022 PMC7049256

[msad276-B44] Moreno M, Marinotti O, Krzywinski J, Tadei WP, James AA, Achee NL, Conn JE. Complete mtDNA genomes of *Anopheles darlingi* and an approach to anopheline divergence time. Malar J. 2010:9(1):127. 10.1186/1475-2875-9-127. 20470395 PMC2877063

[msad276-B45] Muto Y, Yokoyama S. Structural insight into RNA recognition motifs: versatile molecular Lego building blocks for biological systems. Wiley Interdiscip Rev RNA. 2012:3(2):229–246. 10.1002/wrna.1107. 22278943

[msad276-B46] Newton ME, Southern DI, Wood RJ. X and Y chromosomes of *Aedes aegypti* (L.) distinguished by Giemsa C-banding. Chromosoma. 1974:49(1):41–49. 10.1007/BF00284986. 4141301

[msad276-B47] Reidenbach KR, Cook S, Bertone MA, Harbach RE, Wiegmann BM, Besansky NJ. Phylogenetic analysis and temporal diversification of mosquitoes (Diptera: Culicidae) based on nuclear genes and morphology. BMC Evol Biol. 2009:9:298. 10.1186/1471-2148-9-298. 20028549 PMC2805638

[msad276-B48] Saccone G . A history of the genetic and molecular identification of genes and their functions controlling insect sex determination. Insect Biochem Mol Biol. 2022:151:103873. 10.1016/j.ibmb.2022.103873. 36400424

[msad276-B49] Salz H, Erickson JW. Sex determination in Drosophila: the view from the top. Fly (Austin). 2010:4(1):60–70. 10.4161/fly.4.1.11277. 20160499 PMC2855772

[msad276-B50] Sarno F, Ruiz MF, Eirin-Lopez JM, Perondini AL, Selivon D, Sanchez L. The gene transformer-2 of *Anastrepha* fruit flies (Diptera, Tephritidae) and its evolution in insects. BMC Evol Biol. 2010:10:140. 10.1186/1471-2148-10-140. 20465812 PMC2885393

[msad276-B51] Shukla JN, Nagaraju J. Doublesex: a conserved downstream gene controlled by diverse upstream regulators. J Genet. 2010:89(3):341–356. 10.1007/s12041-010-0046-6. 20877001

[msad276-B52] Soghigian J, Andreadis TG, Livdahl TP. From ground pools to treeholes: convergent evolution of habitat and phenotype in Aedes mosquitoes. BMC Evol Biol. 2017:17(1):262. 10.1186/s12862-017-1092-y. 29258425 PMC5735545

[msad276-B53] Solovyev V, Balding DJ, Bishop M, Cannings C. In: Balding DJ, Bishop M, Cannings C, editors. Handbook of statistical genetics. Hoboken, NJ, USA: Wiley-Interscience; 2007. p. 1616.

[msad276-B54] Stamatakis A . RAxML version 8: a tool for phylogenetic analysis and post-analysis of large phylogenies. Bioinformatics. 2014:30(9):1312–1313. 10.1093/bioinformatics/btu033. 24451623 PMC3998144

[msad276-B55] Tormey D, Colbourne JK, Mockaitis K, Choi J-H, Lopez J, Burkhart J, Bradshaw W, Holzapfel C. Evolutionary divergence of core and post-translational circadian clock genes in the pitcher-plant mosquito, *Wyeomyia smithii*. BMC Genomics. 2015:16:754. 10.1186/s12864-015-1937-y. 26444857 PMC4594641

[msad276-B56] Toups MA, Hahn MW. Retrogenes reveal the direction of sex-chromosome evolution in mosquitoes. Genetics. 2010:186(2):763–766. 10.1534/genetics.110.118794. 20660646 PMC2954464

[msad276-B57] Verhulst EC, van de Zande L. Double nexus–Doublesex is the connecting element in sex determination. Brief Funct Genomics. 2015:14(6):396–406. 10.1093/bfgp/elv005. 25797692 PMC4652034

[msad276-B58] Wang J, Youkharibache P, Marchler-Bauer A, Lanczycki C, Zhang D, Lu S, Madej T, Marchler GH, Cheng T, Chong LC, et al Icn3d: from web-based 3D viewer to structural analysis tool in batch mode. Front Mol Biosci. 2022:9:831740. 10.3389/fmolb.2022.831740. 35252351 PMC8892267

[msad276-B59] Wang J, Youkharibache P, Zhang D, Lanczycki CJ, Geer RC, Madej T, Phan L, Ward M, Lu S, Marchler GH, et al Icn3d, a web-based 3D viewer for sharing 1D/2D/3D representations of biomolecular structures. Bioinformatics. 2020:36(1):131–135. 10.1093/bioinformatics/btz502. 31218344 PMC6956771

[msad276-B60] Whelan S, Goldman N. A general empirical model of protein evolution derived from multiple protein families using a maximum-likelihood approach. Mol Biol Evol. 2001:18(5):691–699. 10.1093/oxfordjournals.molbev.a003851. 11319253

[msad276-B61] Zadra N, Rizzoli A, Rota-Stabelli O. Chronological incongruences between mitochondrial and nuclear phylogenies of *Aedes* mosquitoes. Life. 2021:11(3):181. 10.3390/life11030181. 33669100 PMC7996624

[msad276-B62] Zhao Y, Jin B, Liu P, Xiao X, Cai L, Xie Z, Kong L, Liu T, Yang W, Wu Y, et al The AalNix3&4 isoform is required and sufficient to convert *Aedes albopictus* females into males. PLoS Genet. 2022:18(6):e1010280. 10.1371/journal.pgen.1010280. 35737710 PMC9258803

